# A Semi-Empirical Approach to Gas Flow Velocity Measurement by Means of the Thermal Time-of-Flight Method

**DOI:** 10.3390/s21175679

**Published:** 2021-08-24

**Authors:** Jacek Sobczyk, Andrzej Rachalski, Waldemar Wodziak

**Affiliations:** Strata Mechanics Research Institute, Polish Academy of Sciences, Reymonta 27, 30-059 Krakow, Poland; rachalski@imgpan.pl (A.R.); wodziak@imgpan.pl (W.W.)

**Keywords:** thermal anemometer, thermal time-of-flight (TTOF), thermal wave, low flow velocity

## Abstract

This paper presents a method of measuring gas flow velocity based on the thermal time-of-flight method. The essence of the solution is an analysis of the time shift and the shape of voltage signals at the transmitter and at a temperature wave detector. The measurements used a probe composed of a wave transmitter and a detector, both in the form of thin tungsten wires. A rectangular signal was used at the wave transmitter. The time-of-flight of the wave was determined on the basis of the time shift of two selected characteristic points of the voltage waveform at the transmitter and the wave detector. To obtain the correct velocity indication, a correction in the form of a simple power function was applied. From the measurements performed, the relative uncertainty of the method was obtained, from approx. 4% of the measured value at an inflow velocity of 6.5 cm/s to 1% for an inflow velocity of 50 cm/s and higher.

## 1. Introduction

### 1.1. Introduction to the Thermal Time-of-Flight Method

Measurements of the gas flow velocity constitute an important branch of metrology. They are widely used in industrial and laboratory measurements. The need to measure gas flow velocity appears in the chemical, aviation, and automotive industries, in the control of ventilation systems. Another issue is the study of air flows in closed spaces (such as production halls, offices, storage rooms, etc.). There is a great variety in the methods of measuring the gas flow velocity.

In simplified terms, the following types of methods of measuring the gas flow velocity can be distinguished (with exemplary devices):

- Based on pressure measurement (damming tubes, orifices, nozzles);
- Mechanical (vane anemometers);
- Thermal (hot-wire anemometers);
- Marker (LDA, PIV);
- Ultrasonic.

The thermal time-of-flight method uses a heated volume of flowing fluid as a marker. Its advantages and disadvantages are best presented against the background of other methods. The most important advantage is the ability to measure very low flow velocities, even in the order of mm/s, i.e., in the range where pressure-based and mechanical anemometers cannot be used. Compared to other marker methods, it does not require the introduction of a foreign phase (solid or liquid) into the flow, so it is minimally invasive. Another advantage of this method is its low sensitivity to changes in temperature or the composition of the flowing gas, i.e., in conditions where the use of hot-wire anemometers is difficult or even impossible. It requires neither complicated nor expensive apparatus such as LDA and PIV methods. The main drawback of thermal time-of-flight method is the low bandwidth determined by the time of the marker’s flight, which distinguishes it negatively from other methods. The spatial resolution of the velocity measurement, although better than that of mechanical anemometers, is inferior to that of hot-wire anemometers due to the complexity of the probe, which must include a wave transmitter and detector. Due to the fact that the speed is determined on the path between the transmitter and the wave detector, the correct orientation of the probe in the measured flow is important, therefore it is difficult to use it in conditions where the velocity direction changes during the measurement.

### 1.2. Motivation of the Study

Interest in the thermal time-of-flight method and work on its development stem from the need to measure very low gas flow velocities, in conditions where the gas composition and temperature are unknown or change during the measurement. The second reason is the need to conduct such measurements in real conditions (outside the laboratory), where flows are turbulent.

This paper describes an attempt to modify the thermal wave method so that it is enough to use only one wave detector to determine the gas flow velocity. The idea is to determine the flow velocity on the basis of an analysis of the time intervals between the characteristic points of the voltage waveforms at the temperature transmitter and the detector. This simple approach, although more empirical than physical by nature, is in fact a step forward in meeting the needs mentioned above.

## 2. Materials and Methods

### 2.1. Basics of the Thermal Time-of-Flight Method

The basis for determining the velocity of a flowing gas stream using the thermal wave method is the measurement of the temperature wave propagation time in the tested flow over a known distance. The test probe comprises a wave transmitter, usually in the form of a fine wire, and one or two wave detectors (resistance thermometers), also in the form of thin wires, positioned downstream of the transmitter. An important issue is the method of determining the time-of-flight of the wave. Two interconnected phenomena are involved in the process of propagation of a temperature wave in flowing gas: Wave drift with the flow velocity, and thermal diffusion. For a sinusoidal wave, the dependence of the wave phase velocity on the flow velocity is described by the relationship [1,2]:

(1) $$v_{T}=v\sqrt{1+\frac{4\kappa^{2}\omega^{2}}{v^{4}}},$$

where *v_T_* is the temperature wave phase velocity (m/s), *v* is the gas velocity (m/s), *ω* is the wave frequency (rad/s), and *κ* is the gas temperature diffusivity (m^2^/s). From Equation (1), it follows that the velocity *v_T_* of the temperature wave is always greater than the drift velocity *v* and depends on the frequency of the wave. If in Formula (1) the fraction is negligibly small, which can be expressed in terms of the ratio of the Strouhal and Peclet numbers:

(2) $$\frac{Sr}{Pe}=\frac{\kappa \omega }{v^{2}}\ll 1,$$

then, the phenomenon of temperature diffusion can be ignored and the phase velocity of the thermal wave can be assumed to be equal to the flow velocity, i.e., *v* = *v_T_*. Additionally, if the thermal diffusion is negligible, the signal does not change its shape. In this case, it is enough to measure the time interval between any chosen characteristic point of the signal waveform registered by the detectors. The above analysis can be applied to a waveform of any shape—Equation (1) then describes the propagation of the *i*-th harmonic components of the signal with frequencies *ω_i_*.

### 2.2. Main Approaches to the Thermal Time-of-Flight Method

In order to generate a thermal wave in the flowing gas, the temperature of the transmitter must change over time. In the thermal wave method, various types of electric current waveforms heating the wave transmitter are used: It may be a pulse signal [3,4,5,6], a sinusoidal signal [1,2,7,8,9,10] or—easiest to implement and most commonly used—a rectangular signal [11,12]. Other signals that have been used include a pseudo-stochastic signal [13], a signal composed of the sum of sinusoidal waveforms with various appropriately selected frequencies and amplitudes [14], and a rectangular multifrequency binary sequence (MBS) signal [15]. MBS signals have the property that the major part of the signal power is concentrated in several harmonic components [16].

To determine the time-of-flight of the wave, the following solutions are used: A direct method, determination of mutual correlation of signals on the detectors, and calculation of the phase shift of the signals. In the direct method, the time of flight of the wave is determined on the basis of selected characteristic points in the time waveforms of the signals recorded by the detectors. The most frequently selected points are the beginning of the signal rise [17] or the maximum signal [5,10]. The correlation method consists of determining the time interval between the signals using the cross-correlation function [13,18]. The phase shift of the signals is determined using the spectral analysis [1,12,15].

The correct measurement, especially at very low flow velocities, is possible provided that the influence of temperature diffusivity and the phenomenon of wave dispersion on the signal shape is taken into account. A method based on the harmonic analysis of temperature waveforms exists followed by the determination of phase shifts of individual harmonics [12,13,15]. In this method, the flow velocity is determined by matching the measured phase shifts of harmonic components to the theoretically calculated relationship. This method is insensitive to changes in the temperature diffusivity of the gas, and as a consequence, enables the measurement of the flow velocity in non-isothermal flows and flows with a variable composition of the flowing gas. A certain drawback of this method is the need to use two wave detectors. This leads to complications in the measuring probe and increases its sensitivity to aerodynamic flow disturbances.

Even if we determine the transit time of the wave correctly and take into account the temperature diffusion and wave dispersion, a problem arises related to the phenomenon of the so-called aerodynamic shadow formed behind the transmitter and wave detectors placed in the flow [19,20]. This consists of a reduction in the flow velocity behind the obstacle. In fact, we measure the velocity of the flowing gas *v* between the transmitter and the wave detector or between the two detectors, while what interests us is the inflow velocity *v*_∞_. Unfortunately, since the aerodynamic shadow practically coincides with the temperature trace (in which the detector must be placed), it is impossible to completely eliminate the influence of the former on the measurement result by changing the probe geometry [20].

### 2.3. Measurement Stand

Measurements were carried out in a closed-circuit TANPOZ wind tunnel (Strata Mechanics Research Institute of the Polish Academy of Sciences, Krakow, Poland). Its main features are as follows [21]:

- Measurement chamber dimensions: 0.5 × 0.5 × 1.5 m (W × H × L);
- Velocity range: 0.01–62.0 m/s;
- Turbulence level: <0.4%;
- Temperature and relative humidity: Controlled.

Therefore, measurements were carried out under controlled conditions at low and very low inflow velocities. The inflow velocity was controlled using a Schmidt thermal anemometer (model SS 20.500), whose measurement range is 0.07–2.50 m/s and measurement uncertainty is 1.5% of the indicated value, but not less than 0.07 m/s. Velocities below 0.07 m/s were estimated using the frequency of the wind tunnel fan inverter with similar uncertainty.

The thermal wave anemometer probe was placed close to the center of the measurement chamber, at a sufficient distance from the Schmidt anemometer to avoid the mutual influence of the two devices.

For the generation and detection of temperature waves, a computer-controlled digital anemometer-thermometer (CCC2002) was used [22]. This enables the imposition of various types of voltage signal on the transmitter and the measurement of voltage on wave detectors, which are resistance thermometers. The transmitter and the two wave detectors were made of tungsten wire; 8 μm in diameter and 6 mm in length (transmitter) and 3 μm in diameter and 3 mm in length (detectors). Thermal signal propagation measurements were made for inflow velocities ranging up to 2.5 m/s. The transmitter operated in a constant temperature-anemometer (CTA) system with a rectangular input. The overheating ratio of transmitter wire alternated between 1.0 and 1.8. The wave frequency was 0.25, 0.5 or 1.0 Hz. Each measurement lasted no less than 10 periods of the thermal wave.

Data acquisition from both anemometers was performed with the use of an analog-to-digital converter (ADC) card (16-bit) from the NI and NI Signal Express software. The voltage range was set to 0–10 V. The resulting resolution was 0.15 mV. The sampling rate of the card was set to 10 kHz per channel. In order to keep the level of signal-to-noise ratio (S/N) as high as possible the measuring system was connected to an “on-line” uninterruptible power supply (UPS). This type of UPS isolates connected devices from the mains and its disturbances.

**Figure 1:**
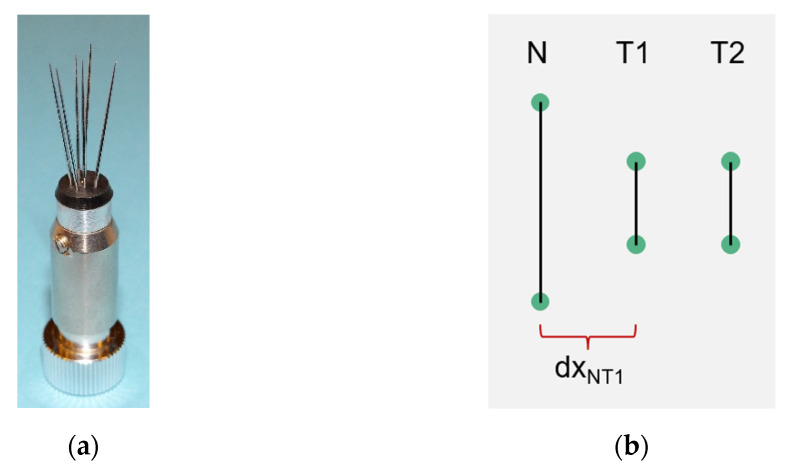
Photograph of the thermal wave anemometer probe (**a**) and a diagram of its geometric configuration (**b**). The wires (invisible on the photograph and shown on the diagram as black lines) are welded at the tips of the supports.

The geometric configuration of the probe is shown in Figure 1b. The distance of the detector T1 from the transmitter N (denoted as *dx_NT_*_1_) was 3.7 mm.

### 2.4. Data Analysis and Visualisation

To analyse and visualize data, the OriginLab OriginPro software was used. All the presented analyses were done by hand in order to track all the phenomena that can be distinguished in the signals. Localizations of the characteristic points (vide Section 3.2) were estimated with the uncertainty ranging from ±0.0012 s for the lowest inflow velocities to ±0.0001 s for inflow velocities equal to 0.4 m/s and higher. These values resulted from the S/N ratio of the detector T1 voltage signal and the sampling rate of the ADC card.

### 2.5. Shapes of Recorded Voltage Waveforms

**Figure 2:**
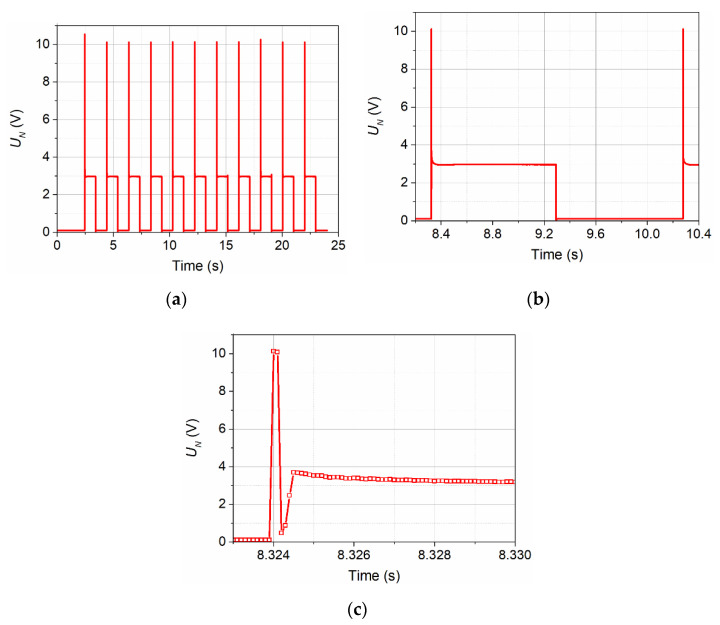
Voltage signal supplied to the transmitter by the CCC2002 device: course of 11 consecutive pulses (**a**), one selected pulse (**b**), and structure of the overdrive (**c**).

The voltage signal supplied to the transmitter of the thermal wave N had a shape similar to a rectangle. Figure 2a shows the course of 11 consecutive pulses, while Figure 2b shows one selected pulse.

The overdrive visible at the beginning of each pulse is to obtain the steepest possible edge of the signal heating the transmitter. The structure of this overdrive is shown in Figure 2c.

Detectors T1 and T2 working in thermometer mode react to temperature changes in their surroundings. The voltage signal recorded on the resistance bridge of each of the detectors is proportional to this temperature. The thermal wave propagating from the transmitter to the detectors quickly weakens over time. Therefore, the signal recorded by detector T2 is usually much weaker than that recorded by detector T1.

**Figure 3:**
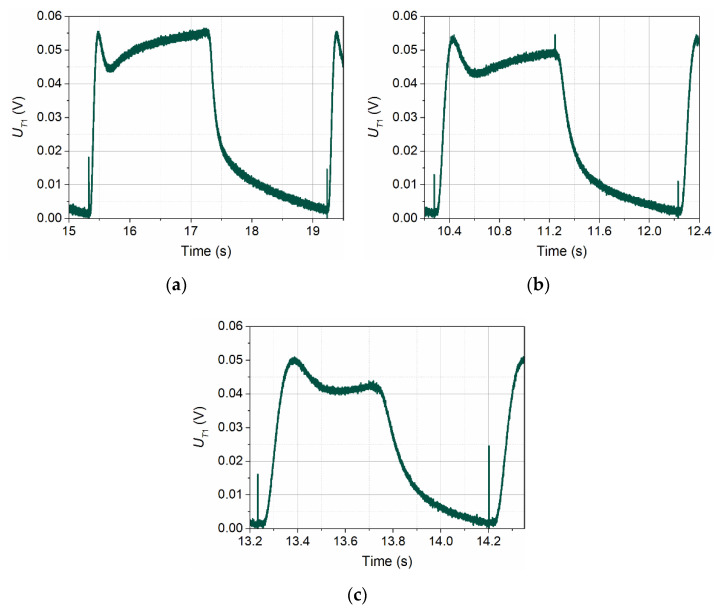
Shapes of voltage signals recorded by detector T1 in conditions of no flow for three different frequencies of the transmitter wave: *f* = 0.25 Hz (**a**), *f* = 0.50 Hz (**b**), and *f* = 1.00 Hz (**c**).

Figure 3 shows the shapes of voltage signals recorded by detector T1 in conditions of no flow (*v*_∞_ = 0 m/s,) for three wave frequencies: *f* = 0.25 Hz, *f* = 0.50 Hz, and *f* = 1.00 Hz.

In all three signals presented in Figure 3, one can distinguish the rising edge of the signal, the peak related to the voltage signal overdrive at the transmitter, the beginning of a further slow increase of the signal, and finally fall of the signal. The differences in the shapes of these three pulses are due to the different lengths of the thermal wave period. The change in the shape of the pulses towards sawtooth with the increase in the frequency of the wave is caused by the shorter heating time of the transmitter in the successive periods of the thermal wave. As a result, the fragment of pulses related to the heating of the transmitter supports is shortened. Further increasing the frequency of the thermal wave would shorten this fragment further until the waveform was very close to the sawtooth. At the same time, the amplitude of this waveform would be further reduced.

A very narrow peak (pin) visible just before the beginning of the signal growth recorded by detector T1 is formed when the transmitter is overdriven. It is the result of crosstalk between the channels of the ADC card’s multiplexer or is caused by the transmission of an electromagnetic wave between the transmitter and detector.

**Figure 4:**
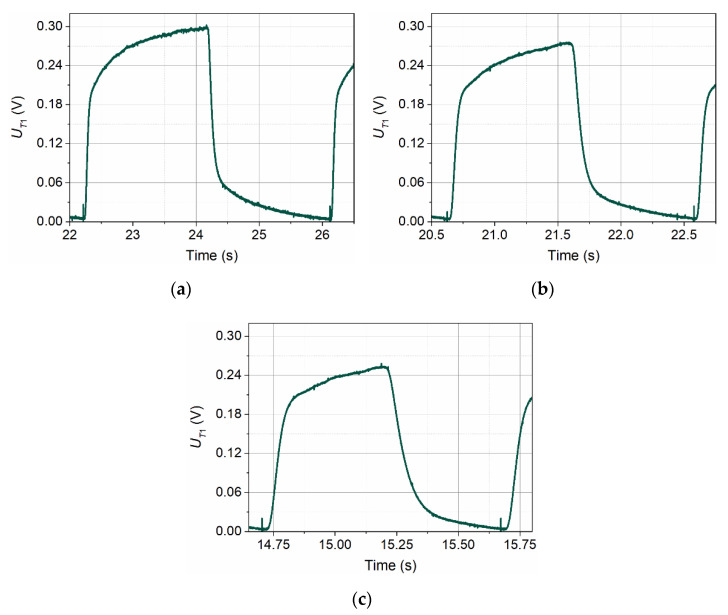
Pulse shapes recorded by detector T1 for three thermal wave frequencies: *f* = 0.25 Hz (**a**), *f* = 0.50 Hz (**b**), and *f* = 1.00 Hz (**c**), at the inflow velocity *v*_∞_ = 0.065 m/s.

In the case when the inflow velocity is non-zero, the amplitude of the signal on detector T1 increases and its shape changes. This is illustrated in Figure 4. The change of the signal shape is related to two phenomena. The first is the transport of the heated medium towards the detectors. This transport shortens the time between the generation of the thermal wave and the moment of its detection, which reduces the level of thermal energy dissipation and increases the temperature recorded by the detectors. The second phenomenon is the increased transfer of thermal energy from the transmitter to the medium due to cooling. The flow that cools the transmitter also changes the nature of the voltage–current relationship, although its actual temperature does not change significantly, since the transmitter operates in a constant temperature system (CTA).

The graphs of pulses from the T1 detector presented in Figure 4 were recorded at an inflow velocity of *v*_∞_ = 0.065 m/s. They differ from those shown in Figure 3 in having more than five times greater amplitude and no initial peak. A further increase in the inflow velocity causes the shape of the pulses recorded by detector T1 to approach a rectangular shape.

**Figure 5:**
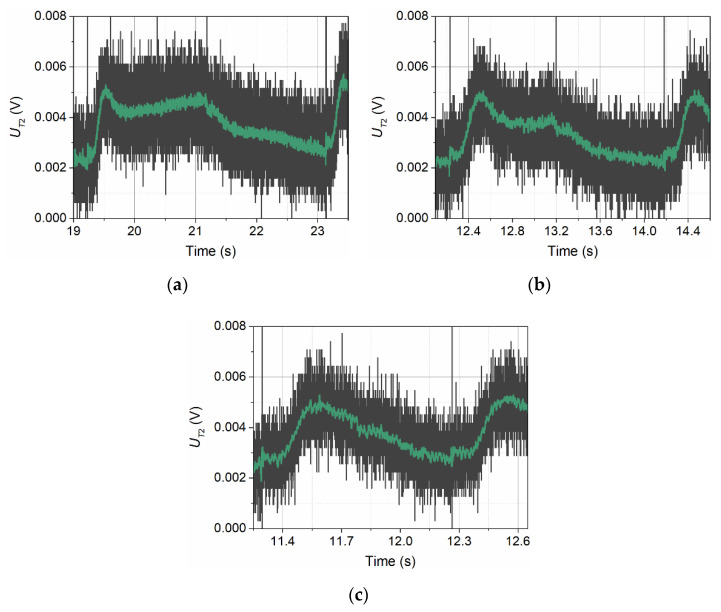
Shapes of voltage signals recorded by detector T2 in conditions of no flow and for three thermal wave frequencies: *f* = 0.25 Hz (**a**), *f* = 0.50 Hz (**b**), and *f* = 1.00 Hz (**c**).

Figure 5 shows the signals recorded by detector T2 at *v*_∞_ = 0.0 m/s. The thermal signal reaching detector T2 is greatly weakened in the absence of forced convection. This is mainly due to the distance of detector T2 from the transmitter, but also due to the fact that the signal encounters an obstacle in its path in the form of detector T1. In Figure 5, the thermal signal is marked in dark grey. The nature of the graphs indicates that they were recorded at the limit of ADC card resolution. The amplitude of these signals does not exceed a few mV at the measuring range of 10 V. The curves created by smoothing the measured waveforms are marked in green. Signals recorded by detector T2 resemble the corresponding signals recorded by detector T1, but have a much lower amplitude. For the thermal wave frequency *f* = 1 Hz, the signal has a sawtooth shape.

**Figure 6:**
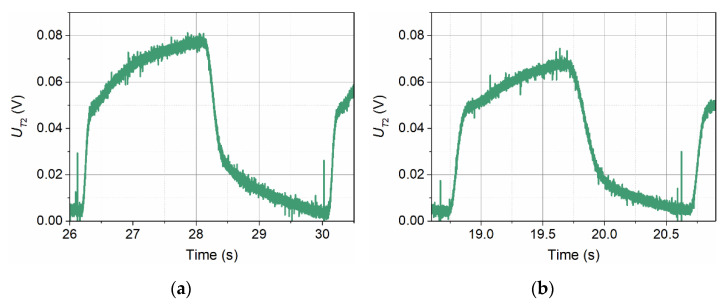

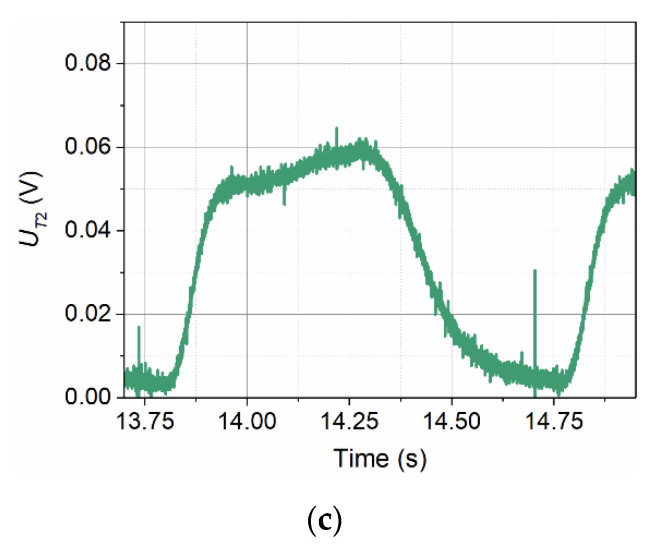
Pulse shapes recorded by detector T2 for three thermal wave frequencies: *f* = 0.25 Hz (**a**), *f* = 0.50 Hz (**b**), and *f* = 1.00 Hz (**c**), at the inflow velocity *v*_∞_ = 0.065 m/s.

At an inflow velocity of *v*_∞_ = 0.065 m/s, as in the case of detector T1, the amplitude of the signals recorded by detector T2 increases significantly (Figure 6). In this case, however, it increases by more than an order of magnitude. The shape of the pulses also changes in a similar way.

**Figure 7:**
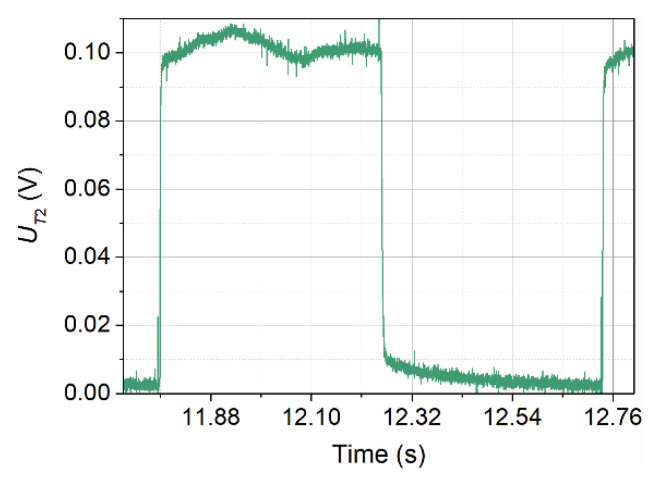
The shape of the pulse recorded by detector T2 for the thermal wave frequency *f* = 1 Hz and the inflow velocity *v*_∞_ = 1.97 m/s.

An increase in the inflow velocity to *v*_∞_ = 1.97 m/s results in a change in the voltage waveform recorded by detector T2 (Figure 7). It shows a further, though relatively small, increase in the signal amplitude, a significant change in shape towards rectangular, and a disturbance occurring in the area of maximum voltage values.

The pulse shapes are adversely affected by aerodynamic disturbances caused by the presence of the transmitter and detector T1. The influence of aerodynamic disturbances from the transmitter was visible in Figure 4, where the waveform in the area of maximum voltage values showed a slight undulation. In the case of detector T2, standing behind two obstacles in the form of pairs of supports with stretched resistance wires, this disturbance is more visible [23,24].

## 3. Results and Discussion

### 3.1. Characteristic Points of Recorded Voltage Signals

**Figure 8:**
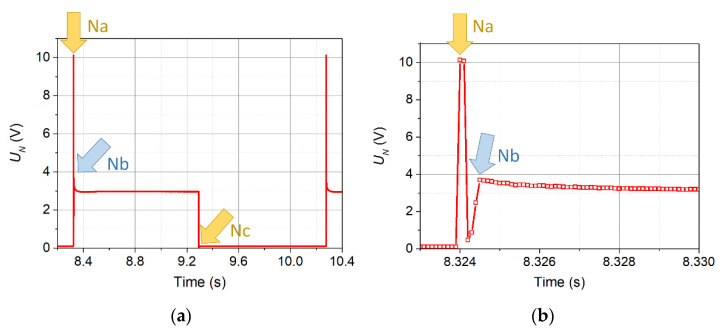
Selected characteristic points of the voltage signal provided at the transmitter (**a**). Precise position of point Nb (**b**).

The proposed method of velocity measurement consists of determining the time interval between the characteristic points of the voltage waveform on the wave transmitter and detector. Figure 8 shows the signal from the transmitter. It essentially has only two characteristic points—marked Na and Nc. The third (Nb) results only from the initial overdrive.

**Figure 9:**
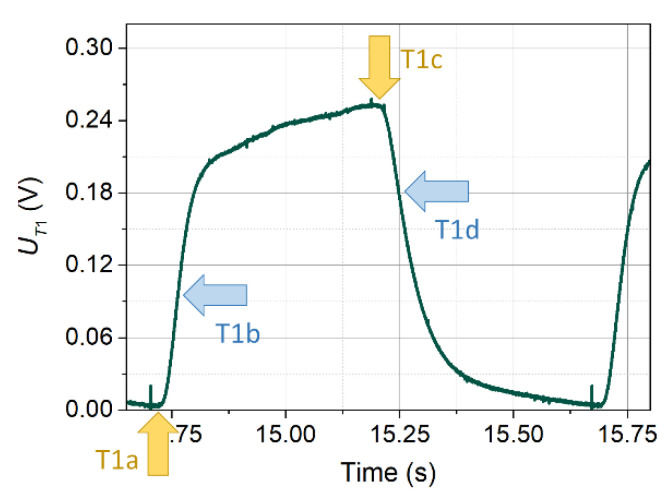
Selected characteristic points of signals from the detectors, using the example of the signal from detector T1 for the inflow velocity *v*_∞_ = 0.065 m/s and the wave frequency *f* = 1.0 Hz.

In the case of signals recorded by detectors, due to their complex shape, several characteristic points can be distinguished. Due to the change in the shape of the signal as a function of the inflow velocity, not all points can be determined for each inflow velocity. Four points (from T1a to T1d), the easiest to determine with the use of automatic methods, were selected for further analysis. They are shown in Figure 9, taking as an example the signal from detector T1 for the inflow velocity *v*_∞_ = 0.065 m/s and the wave frequency *f* = 1.0 Hz. The points T1a and T1c correspond to points Na and Nc, respectively, on the transmitter, and points T1b and T1d are the inflection points of the rising and falling signal portions, respectively.

Determination of the moment of the characteristic point occurrence can be done in many ways. For the binary transmitter signal, simple thresholding may be just enough. However, detectors signals require more sophisticated handling. One of the simplest approaches starts with the calculation of derivative of the voltage signal together with the appropriately chosen smoothing. The derivative amplifies changes in the original waveform while “ignoring” the constant values. This enables a much easier determination of all chosen characteristic points in the detectors signals by hand. Unfortunately, precise automatic detection of points T1a, T2a, T1c, and T2c may be difficult and in certain cases even impossible.

**Figure 10:**
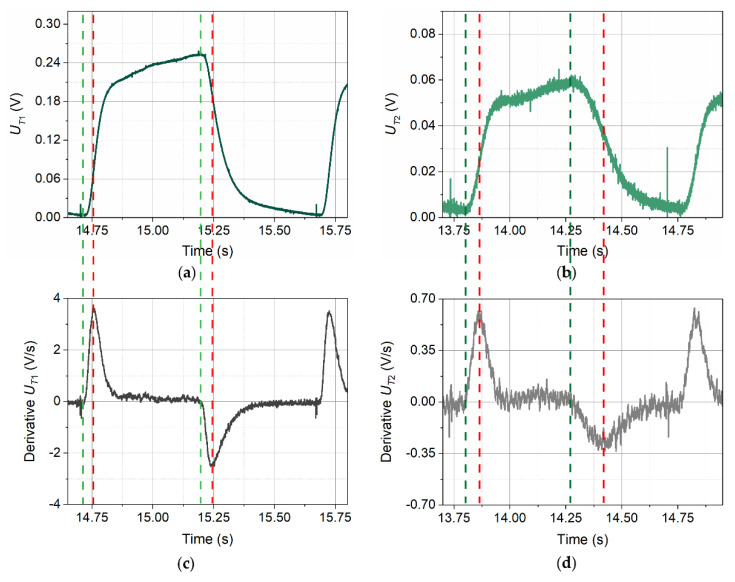
Pulses recorded by detector T1 (**a**) and T2 (**b**) at the thermal wave frequency *f* = 1.00 Hz, and the inflow velocity *v*_∞_ = 0.065 m/s and their derivatives, (**c**,**d**), respectively. Dashed lines connect characteristic points from the corresponding graphs.

On the other hand, the four remaining points are easy to be determined by simply finding the locations of peaks of the derivatives (Figure 10).

Derivatives presented in Figure 10c,d despite the preliminary smoothing are still “jagged”—exhibit some noise. Further smoothing would make them less noisy, but the stronger the smoothing, the more the peak positions will be shifted. Therefore, in order to find positions of peaks accurately it is indispensable to apply approximation with a function. In this study, the best results (the lowest χ^2^ values) were obtained using the Gumbel probability density function (3) [25,26,27], which was chosen only due to its shape:

(3) $$U=U_{0}+A\;exp\left\{-exp\left[-\left(\frac{t-t_{c}}{w}\right)\right]-\left(\frac{t-t_{c}}{w}\right)+1\right\},$$

where *U* is the voltage (V), *U*_0_ is the voltage offset (V), *A* is the amplitude (V), *t* is the time (s), *t_c_* is the centre of the peak (s), and *w* is the peak’s width (s). Fits were made with the use of nonlinear estimation (Levenberg–Marquardt algorithm [28,29]).

The procedure described here is very precise—the expected accuracy for signals with sufficient S/N value is better than the time resolution resulting from the ADC sampling rate.

### 3.2. Selection of Characteristic Points

**Figure 11:**
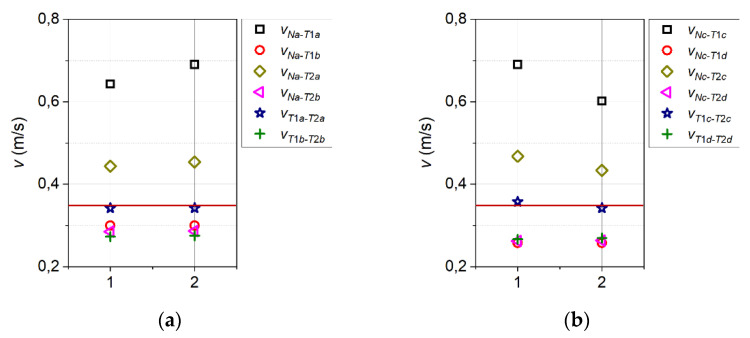
Velocity maps made with the use of selected characteristic points related to the voltage signals rising (**a**) and falling (**b**) edges. For clarity, velocities resulting from pairs of points not corresponding to each other were omitted. The same applies to pairs containing point Nb. The numbers along the horizontal axes indicate whether the data columns correspond to the first or second thermal wave period.

Analysis of the time intervals between the characteristic points of the corresponding voltage pulses from the transmitter and both detectors, with knowledge of the actual distances between these three elements, enables the preparation of a velocity map (Figure 11). The values determined are the velocities resulting from the reference of time to distance. The time intervals were determined by pairing the characteristic points on the voltage waveforms of the transmitter N and the detectors T1 and T2 within the same period of the thermal wave. The analysis was performed for two distant periods of the thermal wave in order to check the repeatability of the results.

In Figure 11, the horizontal red line marks the inflow velocity *v*_∞_ = 0.352 m/s. The velocity values determined by pairing characteristic points obtained from voltage waveforms from the detectors (the pairs T1a-T2a and T1c-T2c) are closest to the set inflow velocity. However, the main limitation of the velocity determination method based on information from both detectors is the requirement of steady flow. Even slight changes in the flow direction lead to a deterioration or even loss of the signal at detector T2.

If all points related to detector T2 are removed from the graphs in Figure 11, only four series of points will remain. The first two series, referring to the characteristic points Na-T1a and Nc-T1c, give velocity values that are around twice as high as the correct values, and fails to ensure repeatability of the results. In the other two series, two points relate to the pair of characteristic points Na-T1b (in Figure 11 they correspond to values closer to the inflow velocity), and two relate to the pair of characteristic points Nc-T1d (in Figure 11 they correspond to values further from the inflow velocity). Therefore, the characteristic points Na-T1b were selected for further considerations.

### 3.3. The Method of Correcting the Velocity Measurement Results

**Figure 12:**
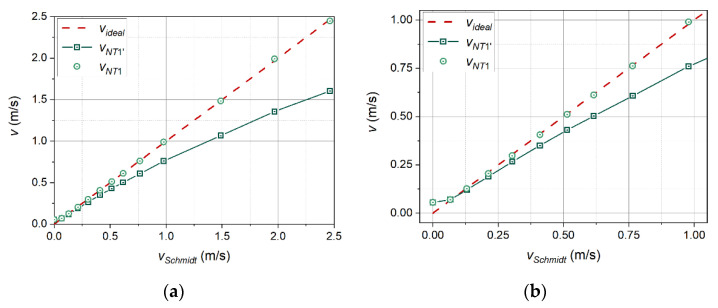
Velocity measurement results without correction (squares), ideal relationship (dashed line), measurement results after applying the correction (circles): all the results (**a**), and a subset of the results limited to velocities below 1 m/s (**b**).

An analysis was made of measurement data obtained in a cycle of 13 measurements for a velocity range from 0.0 to 2.5 m/s. Only one thermal wave frequency, *f* = 1.0 Hz, was taken into account. The calculations of every single velocity value used three adjacent periods of the thermal wave occurring not earlier than five periods from the moment of activation of the transmitter. The obtained results, relating to the characteristic points Na-T1b, were averaged to obtain one velocity value. The results are shown in Figure 12a and are marked with green squares. Figure 12b shows a subset of these results limited to velocities below 1 m/s, to better visualise their nature in the range of lowest velocities.

Figure 12 compares the inflow velocity measured with the Schmidt anemometer (horizontal axis) with the velocity determined with the thermal wave anemometer (vertical axis) using the method described above. The figure also includes a dashed line that shows the ideal relationship of the two velocities (ratio 1:1). If the velocity values determined with the use of the thermal wave anemometer were close to the actual values, the measurement points (squares) should appear on this straight line.

**Figure 13:**
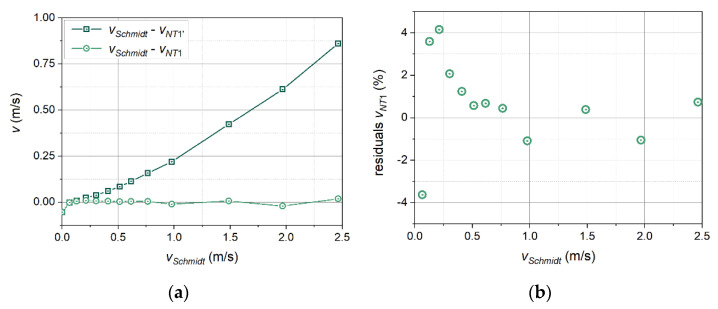
Differences in velocity values between measurements and inflow velocity (**a**) and residuals of measurement with correction (**b**). The measurement results are marked with squares, and the measurement results after applying the correction are marked with circles.

Figure 12 shows that the higher the inflow velocity, the greater the difference between the results of the measurements using the thermal wave anemometer and the actual values. The presentation of these deviations as a function of the velocity in the wind tunnel reveals their exponential character (Figure 13a). The imposition of a correction in the form of a power function (4) of Belehradek type [30] on the measurement results leads to a significant improvement in the indications of the thermal wave anemometer. Adjustment of the values of parameters *a* and *n* require only a few iterations. Measurement points with the applied correction are marked in Figure 12 and Figure 13 with circles.

(4) $$v_{NT1}=v_{NT1^{\prime }}+a\cdot {\left(v_{NT1^{\prime }}-v_{p}\right)}^{n}$$

Designations used:

- *v_NT_*_1_—velocity with applied correction;
- *v_NT_*_1′_—measured velocity;
- *v_p_*—velocity of thermal wave propagation in the current medium;
- *a*, *n*—parameters of the Belehradek function, which are determined during fitting.

Determined values of the parameters of Equation (4):

- *a* = 0.409;
- *n* = 1.650;
- *v_p_* = 0.055 m/s.

Figure 13b shows a graph of the relative differences (expressed as a percentage value) between the velocity values corrected using the power function and the set values in the wind tunnel. Deviations of the corrected velocity values from the set values amount to about 1% for velocities above 0.5 m/s, and increase to about 4% with a decrease in velocity. The increase is systematic, hence it will be possible to apply a simple second-order correction to reduce the value of the deviations in the range of lowest velocities. The higher deviations in the range of lowest velocities may also result from the metrological properties of the instrument used as a reference, the Schmidt anemometer.

In the graphs in Figure 12 and Figure 13a, one point is visible with behaviour different from the others. This point was determined in the absence of inflow (*v*_∞_ = 0 m/s). It was placed on these graphs since the related velocity value appears in Equation (4) as the parameter *v_p_*. In practice, the value of this parameter should be determined at the beginning of each measurement (since it is the velocity of thermal wave propagation in the current medium), and then the actual measurement should be carried out with discrimination against signals with such low amplitudes (see Figure 3). Otherwise, measurements at velocities close to the thermal wave propagation velocity *v_p_* and lower will carry greater measurement uncertainty.

## 4. Conclusions

In light of the presented results, one can draw the following conclusions:

- The estimated thermal time-of-flight value strongly depends on the chosen characteristic points;
- for the single detector probe the most accurate flow velocity estimations can be achieved with the use of the following points: The beginning of the transmitter signal rise (point Na) and the inflection point of the detector signal rising slope (point T1b);
- flow velocity values resulting purely from the thermal time-of-flight estimations (with the use of Na-T1b pair of characteristic points) vary from the inflow velocity, and the difference increases with the increasing velocity;
- application of a simple numerical correction transfers the achieved results into an acceptable region.

The main features of the described measurement method are as follows:

- It does not require the use of information provided by detector T2;
- The algorithm calculating flow velocity values directly from the thermal time-of-flight estimations requires only two arguments—the positions of two points, Na and T1b. Moreover, only the position of T1b depends on flow;
- Of all the characteristic points of the signal from detector T1, T1b is the easiest to determine with the use of automatic methods. Moreover, its determination is the most unambiguous and accurate.

Further research will be carried out for probes with a different spatial arrangement, i.e., with various mutual positions of the transmitter and detector. Work will also aim to improve the accuracy of the method.

## Data Availability

The data presented in this study are available on request from the corresponding author.
